# Understanding disaster resilience in communities affected by recurrent drought in Lesotho and Swaziland—A qualitative study

**DOI:** 10.1371/journal.pone.0212994

**Published:** 2019-03-01

**Authors:** Joseph K. Kamara, Kingsley Agho, Andre M. N. Renzaho

**Affiliations:** 1 School of Social Sciences and Psychology, Western Sydney University, Sydney, New South Wales, Australia; 2 World Vision International, Southern Africa Regional Office, Mbabane, Swaziland; 3 School of Sciences and Health, Western Sydney University, Sydney, New South Wales, Australia; University of California Los Angeles, UNITED STATES

## Abstract

**Background:**

Lesotho and Swaziland experience intense, recurring drought resulting in disaster situations. Despite the recurrence of drought, both its influence on rural subsistence communities’ support systems and the actions that enable structures of resilience remain poorly understood. Each incidence of drought stimulates a disaster resilience discussion that stalls without achievement of positive results until the next disaster. This study has examined the influence of recurring drought on communities’ inherent resilience and proposes an evidence-based framework to enhance community resilience.

**Methods:**

Data were collected from 16 focus group discussions (N = 197) in the Highveld, Midveld and Lowveld agro-ecological regions of Swaziland and Lesotho. Themes and subthemes have been identified, defined, categorised and narrated using structuration theory as a guide.

**Results:**

Resilience activities were found to be characterised by knowledgeability and changes in behaviour in adapting and applying appropriate actions, all of which were enhanced by institutional support. The effectiveness of institutional support hinged on harnessing communities’ knowledge, social groups, value systems, connectedness, participation, decision-making and collectivism.

**Conclusion:**

Large-scale interventions to build and strengthen resilience are urgently needed in order to maintain cohesiveness and development gains, especially because rural subsistence farmers make up the majority of these two countries’ populations. Policies that integrate resilience with national development planning must be urgently developed and executed.

## Introduction

The concept of resilience has been used in various disciplines since the early 18th century [1]. However, its application to ecological discussions only emerged in the early 1960s [2]. Since then, resilience has become foundational in theories of adaptive systems but is still the subject of extensive debate across many research areas [3]. The term ‘resilience’ has no universally accepted definition. Those who attempt to define this term limit its definition to suit their research interests. Walsh-Dilley and Wolford posited that resilience should be defined in relation to what, by whom and for what purpose, so as to enable broad exploration of its grounds of contention [4]. Gallopin [5] defined resilience as “the ability of a multistable system to keep the values of its state variables within a given domain of attraction in the face of perturbations, and is not concerned with the stability or constancy of the state within the basin” (p 298). For this study, resilience is defined as the ability to anticipate, endure, recuperate from and overcome disaster-induced distress [6].

There is growing awareness of resilience as a cornerstone to overcoming natural disasters through improvements in the human absorptive, adaptive and transformative capacities [7]. These capacities function well if they exist within individuals, families and communities prior to the occurrence of a disaster. Absorptive capacity is the ability to undertake carefully planned protective action and to cope with stress; adaptive capacity is the ability to anticipate, prepare and adjust to change for better outcomes; and transformative capacity is the ability to effect changes that reduce exposure to risk [7]. Thus resilience to drought in the context of our study is an evolving outcome of collective responses arising from the interaction of various factors coupled with effective drought risk governance [8].

For the purpose of this study, drought is defined as a prolonged period of below-normal precipitation resulting in crop damage, decreases in stream flow and declining water reservoirs. There are four major categories of drought: meteorological, agricultural, hydrological and socioeconomic drought. Meteorological drought is a deficit of precipitation in comparison to the average expected amount over a given period in an area; agricultural drought is concerned with the effects of meteorological drought on plants at different stages of plant/crop development; hydrological drought refers to insufficient stream flows, reservoir levels and groundwater to meet the prevailing demand for water; and socioeconomic drought refers to the effects of meteorological, agricultural or hydrological droughts on a given community or society [9, 10]. Lesotho and Swaziland experience a combination of the above categories which often starts with meteorological drought.

This study examined resilience to drought in Lesotho and Swaziland. These two African countries are similar politically, geographically, agro-ecologically, economically and demographically. Politically, both monarchies have strong traditional governance. However, Swaziland is an absolute monarchy while Lesotho has a monarchy alongside a parliamentary constitution. Geographically, they are both landlocked and located in the southern Africa region. Lesotho is surrounded by the Republic of South Africa. Swaziland shares borders with Mozambique in the north-east and South Africa in the south, north and west.

Agro-ecologically, the two countries’ vegetation is primarily montane grassland with scattered patches of coniferous forests that have been heavily degraded [11]. The agro-ecological zones are classified as veld. The veld is the vast open grassland of southern Africa usually distinguished by altitude into Highveld, Midveld and Lowveld [12]. The Highveld is mountainous with an elevation between 1200 and 1800 metres above sea level; the Midveld is areas located at intermediate altitudes between 600 and 1200 metres above sea level; while the Lowveld is low-lying terrain with an elevation between 150 and 600 metres above sea level [12]. Regardless of altitude, the veld carries a variety of vegetation and crops, but maize is the main staple grown by over 80% of farmers annually and this crop is highly dependent on rain [13, 14]. All three zones experience drought but its impacts vary due to a host of interacting factors such as access to alternative livelihoods, social deprivation, poverty, levels of land degradation and differences in altitude [15, 16]. For example, the cool and wet temperatures in the Highveld and Midveld sustain crops for longer periods compared to the high temperatures in the semi-arid Lowveld [17]. Additionally, the major centres of economic opportunities other than farming in both countries are located within or in close proximity to the Highveld.

Economically, Lesotho and Swaziland are lower-middle income economies integrated within the BLNS countries. ‘BLNS’ is the collective term used to describe the countries of Botswana, Lesotho, Namibia and Swaziland. The BLNS countries share a common customs union and a common monetary region with South Africa. The BLNS have adopted the South African fiscal policy and their currencies are tied at parity [18]. Almost half of the total revenues of Lesotho and Swaziland come from the South African Customs Union [19, 20]. Over 40% of each country’s population lives below the poverty line of USD 1.25 per day [21]. Demographically, Lesotho has a population of 2 million while Swaziland’s is 1.13 million. Fifty-one per cent of the population is female in both countries [22, 23]. In Lesotho the prime working age group of 25–54 years comprises 38% of the population [24], while 35% of Swaziland’s population falls into this age group [25]. Both governments remain the largest providers of formal employment to their peoples [26, 27]. These similarities between the two countries offer a compelling case for the study of their rural subsistence populations’ resilience and an opportunity to acquire reliable results that can be applied across the southern Africa region.

We have focused on resilience to drought in these two countries for multiple reasons, including the frequency of droughts, the severity of their socioeconomic impacts, the similarities between the two countries and their large, rural subsistence farming populations. Firstly, both countries have experienced severe droughts every two to four years since the early 1980s (Table 1). Secondly, recurrent droughts have imposed heavy burdens on their economies. For example, the 2015–16 drought cost the Swaziland government 18.58% of government expenditure, which was equivalent to 7.1% of gross domestic product (GDP) [26]. The same drought cost the Lesotho government up to 7% of GDP for drought mitigation interventions [28]. In addition, the 2015–16 drought left 35% and 32% of the populations in Lesotho and Swaziland, respectively, in need of humanitarian assistance [16, 29].

**Table 1:** Drought incidence in Lesotho and Swaziland, 1980–2017.

Reference	Country and drought period		Mortality rate/10,000		Emergency thresholds/10,000		Remarks
	Country	Drought	CMR	<5MR	CMR	<5MR	
[16, 29–33]	Lesotho	1981–83	0.4–0.4	3.1–2.9	0.8	2.1	Drought impacts were aggravated by severe land degradation.
		1990–92	0.3–0.3	2.4–2.3	0.8	2.1	
		2001–03	0.5–0.5	3.2–3.3	0.8	2.1	
		2007–08	0.5–0.5	3.3–3.2	0.8	2.1	
		2009–13	0.4–0.4	3.0–2.7	0.8	2.1	
		2015–16	2.5–2.2	9.3–8.9	0.8	2.1	
[16, 29–33]	Swaziland	1981	0.3	3.0	0.8	2.1	The persistently high <5MR could reflect the country’s weak drought resilience.
		1984	0.3	2.5	0.8	2.1	
		1990	0.3	2.0	0.8	2.1	
		1994–95	0.3–0.3	2.3–2.4	0.8	2.1	
		2001–03	0.4–0.4	3.4–3.5	0.8	2.1	
		2006–07	0.4–0.4	3.4–3.2	0.8	2.1	
		2011–13	0.4–0.4	2.4–2.2	0.8	2.1	
		2015–16	1.6–1.6	5.8–5.6	0.8	2.1	

CMR denotes crude mortality rate; <5MR denotes under five mortality rate

Analysis of secondary data benchmarked against the Sphere minimum standards thresholds suggest a high burden of mortality among children aged less than 5 years during drought periods (Table 1) [34,35,36].

Thirdly, the many similarities between the two countries make them comparable for exploring a contextual, evidence-based resilience framework that emerges from the findings. Finally, over 70% of the populations in both countries are comprised of poor, rural subsistence farmers [26, 27]. Recurrent drought destroys their livelihoods and sends them into further poverty. Drought also negatively affects employment opportunities, energy generation, health, nutrition and food security [37].

Despite the recurrence of drought, both its influence on rural subsistence communities’ support systems and the actions that enable structures of resilience remain poorly understood. Therefore, exploring resilience to drought or its lack thereof among the vast majority of the two countries’ populations is an important contribution to benchmarking interventions and development planning, and to the quest to achieve the Sustainable Development Goals (SDGs). The SDGs are 17 global goals for sustainable development agreed upon by member states of the United Nations during the UN general assembly in 2015. Resilience is a critical pillar of the SDGs; there are 25 targets in 10 SDGs that relate to resilience and disaster risk reduction (DRR) [38]. Resilience and DRR are interwoven but independent concepts in disaster management. Resilience refers to the human ability to recover from and overcome the effects of disasters, while DRR is concerned with the identification and analysis of the underlying causes of disasters [39].

Effective disaster management encompasses prevention, preparedness, response and recovery. Early warning systems are a fundamental component of prevention and preparedness that contribute to strengthening community resilience and reducing loss through the prevention of new disaster risks, reduction of existing disaster risks and management of residual risks [38]. Drought is one of the disaster risks that require preparedness. Evidence suggests that neither Swaziland nor Lesotho have effectively prioritised drought resilience even though the early warning system of the Southern Africa Development Committee (SADC) predicts drought frequently [40]. The SADC has well-established drought early warning systems. These include, but are not limited to, the United States government-funded famine early warning systems network (FEWSNET), the Climate Services Centre (previously known as the Drought Monitoring Centre) and the Regional Vulnerability Assessment and Analysis Program (RVAA) [41–43]. Thus the SADC early warning system works in tandem with national meteorological centres and national vulnerability assessment committees to generate, analyse and validate early warning information [42].

This information is shared with the national disaster management authorities (NDMA) in each country, which are mandated by their governments to disseminate these early warnings [44, 45]. The NDMAs use various channels to disseminate early warnings, including print and electronic media, direct sharing of information in bulletins and/or meetings with stakeholders such as NGOs, government ministries and local government administrations [46, 47]. Swaziland and Lesotho have one main agricultural season from October to March. Drought early warning information is gathered, analysed and disseminated before the season, and updates are provided continuously during the season. The NDMAs in Swaziland and Lesotho fall under their respective Prime Ministers’ offices.

The availability and timely dissemination of early warnings enable communities to prepare themselves for impending hazards. However, when early warnings are absent, inaccurate or delivered late, communities remain unprepared and vulnerable to the devastating consequences of the hazard, and require relief intervention.

Since the early 1980s disaster relief interventions, mainly in the form of food aid and fodder, have been used to reduce food deficits and support livestock survival [37, 40, 48]. Such interventions are important in addressing communities’ immediate needs, but they are costly. In addition, these interventions are reactive and aim only to support affected communities to cope with the disaster and return to their pre-disaster conditions, without enabling adaptability or considering lessons learnt from previous experience. Both Swaziland and Lesotho have transferred the burden of funding these interventions to the international community [37, 40]. In the context of recurrent disasters, such interventions create a culture of expectation whereby communities expect the same in subsequent occurrences of disaster. To the contrary, communities should explore their potential to adjust to the changes and function effectively [49].

### Measuring disaster resilience of communities

Over the last two decades, various resilience frameworks and models have emerged to guide disaster preparedness, response and recovery [50, 51]. The most prominent ones are: the PEOPLES resilience; the Disaster Resilience of Place (DROP); the Community Resilience Framework for Emergency Management (CRFEM); the International Federation of the Red Cross and Red Crescent Societies (IFRC) Framework for Community Resilience; the Community-Based Resilience Analysis (CoBRA) Framework; and the Department of International Development (DFID) Resilience Framework.

However, these frameworks remain distant from the contexts and cultures of southern Africa, particularly Swaziland and Lesotho. For instance, the PEOPLES framework is intended to measure the disaster resilience of capital assets in the context of earthquakes [52]; this aim is a mismatch with the risks posed by recurrent drought in the impoverished social economic settings of Swaziland and Lesotho. The DROP framework was designed to measure the social resilience of communities in the US context [53]; there are significant differences in social status, culture, governance and hazard exposure between communities in the USA and in Swaziland and Lesotho. The CRFEM framework is premised on the Australian context, suggesting that successful community resilience is dependent upon the pillars of reflection and awareness; connectedness and inclusiveness; economic dynamism and diversity; vibrant culture; democracy and participation; safety and well-being; and environmental sustainability [54]. However, aligning all these pillars in lower-middle income economies with nascent democracy remains a challenge. The IFRC framework focuses on measuring IFRC interventions instead of benchmarking the characteristics of community resilience [55].

Similarly, the CoBRA framework was developed to measure the impacts of interventions in building community resilience especially in the drylands of the Horn of Africa [56]. However, the contextual differences in peace, security, governance, culture and way of life make CoBRA unsuitable for southern Africa. For instance, pastoralism is a way of life in the conflict-prone drylands of the Horn of Africa, unlike in southern Africa. Likewise, the DFID framework was developed to benchmark DFID’s overseas interventions. The framework is premised on the four elements of social context, hazard type, capacity to deal with the hazard and reaction to the hazard [57]. However, it perceives a disaster as a one-off event and does not consider recurring disasters and how their cumulative effects may induce different reactions depending on context. In addition, the framework is not hazard-specific, which is a critical element of robust resilience frameworks [50].

Importantly, none of these frameworks is a gold standard for benchmarking resilience. Moreover, none of them was specifically developed for the southern Africa context. The frameworks are neither hazard-specific nor location-specific. Emerging evidence suggests that robust resilience frameworks should be both context- and hazard-specific [50, 51]. The inapplicability of these frameworks to the southern Africa region is also evidenced by the repetitive application of reactive disaster relief interventions since the early 1980s [48]. Regionally, there has been limited success in efforts to benchmark resilience. A recent systematic review of resilience in southern Africa noted the lack of a contextually and culturally appropriate resilience framework [58]. The review further observed that most of the relevant studies did not exhibit the rigour necessary to qualify as resilience research. For example, there was no clear delineation between community resilience and community DRR efforts. In addition, many of the studies did not meet the quality assessment for resilience measurement [58].

Nationally, there is very limited research output on resilience in Swaziland and Lesotho. The little that exists is constrained by a focus on evaluating specific relief interventions such as water, sanitation and hygiene, and food distribution, and fails to examine the characteristics of resilient communities [58]. As such, the characteristics of community resilience remain poorly understood. Moreover, there has been limited success in attempts to develop local resilience frameworks. For instance, the Lesotho national resilience framework that was initiated in 2014 has remained in draft form and is non-operational [59]. It focuses on macro-level aspects such as climate risk management and governance, and lacks clarity on how these aspects can be brought down into communities [60]. Furthermore, the draft framework perceives resilience only in the context of disaster recovery.

This study bridges three critical gaps. Firstly, it adds to the limited pool of research on resilience in Swaziland and Lesotho. Making this information available will inspire new ideas and inform new resilience perspectives. Secondly, it examines local experience, knowledge, practices and other inherent capacities that inform the structures of community resilience. Thirdly, it proposes a community resilience framework anchored in data gathered from communities affected by recurrent drought. The framework is important because it is hazard- and context-specific, culturally appropriate and it considers the recurring nature of drought, none of which were addressed by previous research. Therefore, the purpose of this study is threefold: to examine how drought early warnings influence rural subsistence farmers’ resilience; to analyse communities’ inherent resilience capacities; and to derive a contextually and culturally appropriate evidence-based framework to benchmark community resilience.

## Methodological approaches

### Theoretical framework

The study was informed by Giddens’ structuration theory of social action, which emphasises the continuous reproduction of structure [61]. The theory is based on the social interaction of human action (agency) with resources, norms, regulations and support systems (structures) in the production and reproduction of actions [62]. In this theory, Giddens perceived society as a complex series of continuous activities that result in institutions. This implies that structures are produced and reproduced in daily activities [63]. The theory presupposes that the status of a community can be understood through the symbiotic duality of its action and structure, which produces and reproduces change. Structure aids and/or limits action, but is also reproduced by the same action it aids or limits [61]. It is this symbiotic duality of structure that has aided our understanding of the studied communities’ resilience through their continuous efforts to cope with and adapt to recurring drought. In addition, the concept of the continuous production/reproduction of structures has contributed to our understanding of these communities’ persistent search for resilience to recurring drought. Furthermore, the concept of the duality of structure has enabled our understanding of support structures such as the institutions that communities draw from in their quest for resilience.

Therefore, structuration theory has permitted us to posit that the past actions of individuals and communities during emergency responses will influence their present state and their subsequent actions, so as to reproduce structures. Nonetheless, human actions are limited by behaviour, capabilities, choices, knowledge and experience. Structuration theory has enabled our understanding of how the communities produce and reproduce structures, which has subsequently informed our outlook on the policy implications.

Consistent with structuration theory, we examined how communities draw on their stocks of knowledge and experience to build and strengthen their resilience to drought [64]. The study has explored factors such as the communication of meaning, the relationships between work, power and control at individual, family, community and society levels, and the inherent capacities to use these factors to build and strengthen resilience. This study begins with a review of the existing literature on resilience, followed by the role of structuration theory framework. Subsequently it presents data from the focus groups, followed by discussion of the findings and the policy implications.

### Study setting

**Fig 1:**
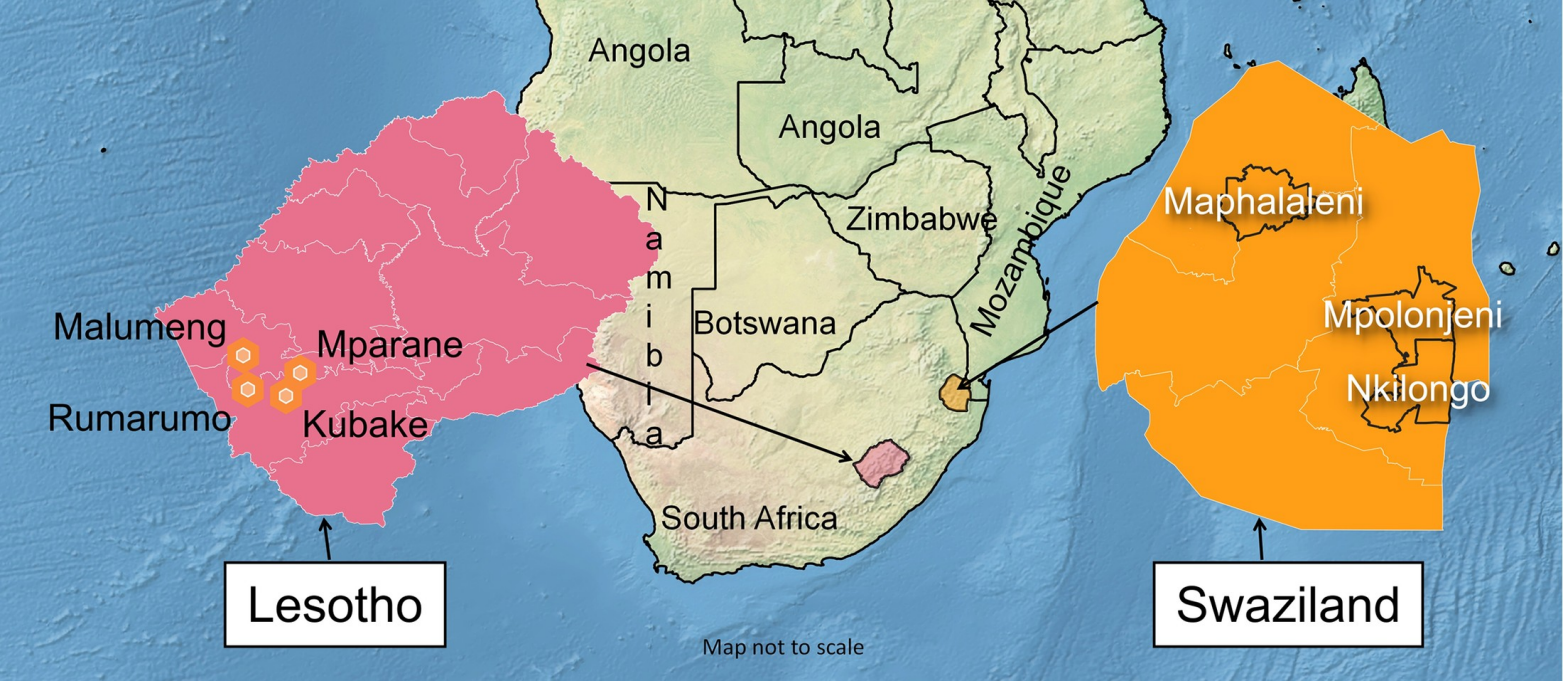
Map of study area. Map source: [65].

This qualitative study was part of a larger study that assessed World Vision’s community-based interventions to reduce poverty and improve health, child protection, resilience, access to services, water, sanitation and hygiene, and disaster preparedness and response in rural Lesotho and Swaziland. The study was conducted in September 2016 in the rural areas of: Nkilongo in the Shiselweni region, Mpolonjeni in the Lubombo region and Maphalaleni in the Hhohho region of Swaziland (Fig 1). The Hhohho region is in the agro-ecological zone of the Highveld, Lubombo is in the Midveld and the Siselweni region is in the Lowveld. In Lesotho, the study was conducted in the Ramarumo, Kubake and Malumeng areas located in the Mafeteng district in the Lowveld agro-ecological zone, and in Mpharane, which is located in Mohale’s Hoek district in the Midveld agro-ecological zone (Fig 1).

### Design and participant recruitment

We used an exploratory qualitative design to understand the communities’ perceptions of disaster resilience. Participants were recruited to focus group discussions (FGDs) from existing social groups including farmers’ associations, women’s groups and community volunteers. There was a high representation of women in our study. This is consistent with their representation in the social groups they were drawn from and with evidence showing that women participate more than men in unpaid work, community events and the informal sector in sub-Saharan Africa [66].

We used FGDs for various reasons. Firstly, FGDs enabled our interaction with participants and also aided the production of new meanings through the hermeneutic effect of participant-to-participant interaction [61]. Secondly, FGDs enabled the capture of collective views and their underlying meanings. Thirdly, FGDs enabled us to gather critical information through group exchanges and the snowballing of ideas. For example, respondent contributions set off a chain of comments that enhanced the quality of discussion. Fourthly, the FGDs were based on existing structures, such as farmers’ groups, and therefore collected rich experiences from those directly impacted by drought. Evidence also suggests that group dynamics have synergistic effects on the generation of study outcomes which would have been missed with other methods [67]. Fifthly, FGDs generated excitement and spontaneous ideas while encouraging every voice to be heard. And lastly, FGDs enabled reflection on the social realities and experiences of each structure nested within structuration theory.

The complexity of the subject matter meant that the sample size could not be predetermined. Moreover, emerging evidence discourages predetermining sample sizes for studies based on FGDs prior to data collection [68, 69]. Therefore, the transcripts from each FGD session were reviewed for emerging concepts prior to conducting subsequent sessions [68]. The review of each FGD session’s outcomes enabled comparison with emerging concepts in subsequent FGDs. By the 16th FGD, many similarities in concepts began to emerge without new information being added. At this point the data collection exercise was ended, as saturation had been obtained with an optimal sample size of 197 participants (Lesotho N = 104; Swaziland N = 93). Each FGD consisted of 5 to 14 participants purposefully drawn from existing social groups consistent with structuration theory.

### FGD process

FGDs were conducted in English by bilingual (English and Siswati; English and Sesotho) interviewers. The FGD facilitators underwent two days’ training followed by a day of practice prior to the exercise. The subject matter of the training included courtesy and ethics, interview techniques, probing, note-taking, confidentiality and group dynamics. FGDs were conducted in areas selected by the participants. Some were conducted in open spaces under trees, while others were carried out in homes selected by the participants. Each FGD was moderated by two facilitators; one moderated the discussion and the second took notes. In addition to the notes, the FGDs were audio-recorded to ensure the information was backed up.

Before the commencement of each FGD, the moderators explained the study objectives. Written and verbal consent to participate, as well as permission to be recorded, was sought and obtained from all participants. Thereafter, ground rules were developed and agreed upon by all participants. For example, participants were encouraged to say what they felt or thought while respecting each other’s views. This included ensuring that only one person talked at a time and, when necessary, participants disagreed respectfully. Participants were reminded that they could opt out of the FGD at any time if they so wished. Furthermore, participants were assured that the information gathered would only be disclosed with their permission, subject to the laws of their countries. Each FGD lasted 90 minutes and saturation was determined when the participants started repeating ideas and no new ideas were being generated. At the end of each FGD session, the main points captured in the notes were read out for participants to make corrections or clarify their points. In addition, the audio files and notes were reviewed by one of two steering committees to establish data saturation and the reliability of the captured information. Audio records were transcribed verbatim before data analysis.

FGDs were based on guidelines informed by existing literature on the subject and by the steering committees. The FGD guide comprised open-ended questions and specific questions to motivate participants to form and articulate their subjective views and experiences on recurrent disasters [70]. Some of the questions included in the guide were: *How do people in your community know that a natural disaster is going to happen*? *What types of disasters has your community experienced over the last three years*? *How have families been affected by these disasters*? *What measures have people in your community taken to recover and bounce back from a disaster*? *What would an ideal recovery from disasters entail*? *What lessons from past disasters has your community implemented*?

### Data analysis

FGD transcripts were independently de-identified and analysed by two researchers, who iteratively read them alongside the field notes to become acquainted with the content [71]. This was followed by a manual coding process based on facts and FGD participants’ responses and views. A code classification tree was created with branches and sub-branches, resulting in a hierarchy of codes that were grouped into relational categories, which were further subdivided into those that belonged to the short term and the long term [72]. The categories were examined for themes and subthemes, which were shared with a third researcher for verification. Differing opinions were resolved by revisiting and discussing the code classification tree until a consensus was reached. The outcomes were presented to the steering committee for feedback on interpretation and for streamlining of themes. The feedback further inspired a thought process to generate a resilience framework rooted in the data. The themes and subthemes are narrated using selected quotations from participants to demonstrate the analytical interpretation while illustrating the findings [73].

### Study governance

The study was overseen by a steering committee in each country. The committee’s mandate involved appraising the FGD guide, advising on the cultural aspects of the FGD process, mobilising communities for the study and advising on how to debrief and feed the study findings back into the study communities. The steering committee in each country comprised World Vision staff, three field coordinators selected from the FGD facilitators, and a consultant. Participation on the committee was voluntary, and the members were trained in basic appraisal and communication skills, as well as skills to facilitate their understanding and ability to fulfill their mandate. The training emphasised awareness of the system of voluntary participation and the confidentiality of discussions.

All data were de-identified before storage to conceal participants’ identities. Electronic copies of the data were password-protected and kept separately from the signed consent forms to reduce the possibility of identification. Permission to carry out the study was given by World Vision’s southern Africa regional office, World Vision Lesotho and World Vision Swaziland. World Vision has long-standing memorandums of understanding with the two national governments through the National Disaster Management Authority in Lesotho and the Ministry of Tinkhundla Administration (the equivalent of a ministry of local government) in Swaziland to collect data as part of the funding service agreements. During data collection, the Tinkhundla headman (head of a constituency) in Swaziland and the executive director of the National Disaster Management Authority in Lesotho were informed about the study and gave their permission for World Vision to carry out the exercise. In addition, the Western Sydney University Human Research Ethics Committee (reference number EX2017/04) approved the study.

## Results

### Demographic characteristics

The study sample was mainly comprised of adults, with 73% female participants and 27% male participants. Participants had mean ages of 47.3 and 52.4 in Swaziland and Lesotho, respectively (Table 2).

**Table 2:** Demographic characteristics of FGD participants.

	**SWAZILAND**								
	FGD1	FGD2	FGD3	FGD4	FGD5	FGD6	FGD7	FGD8	Total
N	16	12	7	15	16	5	10	12	93
M/F	4/12	0/12	1/6	0/15	6/10	0/5	1/9	10/2	22/71
Age: Mean	52.8	45.6	34.1	49.2	47.9	38.6	56.9	53.5	47.3
	**LESOTHO**								
	FGD1	FGD2	FGD3	FGD4	FGD5	FGD6	FGD7	FGD8	Total
N	14	15	7	11	13	13	16	15	104
M/F	6/8	0/15	2/5	5/6	2/11	3/10	8/8	5/10	31/73
Age: Mean	54.9	56.9	49.6	48.7	53.2	48.0	54.9	52.7	52.4

**Swaziland study areas**: Mpolonjeni, Maphalaleni, Nkilongo. **Lesotho study areas**: Mpharene, Ramarumo, Kubake, Malumeng.

### Thematic findings

Consistent with structuration theory, our findings are organised in short/ long term responses based on the context, structure and action at both community and society levels so as to make meaning of the communities’ resilience efforts [74]. Two broad themes demonstrating communities’ adaptation and resilience emerged from the data: the knowledgeability of early warnings and the application of appropriate actions to improve communities’ capacity to withstand and recover from drought. Knowledgeability of early warnings was further subdivided into the two subthemes of making meaning of abstract early warning information and communication of meaning concomitant with structuration theory. The five subthemes that emerged from the application of appropriate actions were: resolve to overcome adversity; community and institutional support; understanding of abstract concepts; exercise of power and control; and determining work relations.

#### Community knowledgeability

We use the term ‘knowledgeability’ to imply that knowledgeable people understand their circumstances and the outcomes of their routines in normal, everyday life, as suggested by structuration theory [61]. The study findings suggest that knowledgeability was demonstrated through:

Making meaning of abstract early warning information: FGD participants in both Lesotho and Swaziland identified various early warning signs of disasters (Table 3). Their understanding of early warnings was based on experience and location. The most common early warnings were based on observation of the environment and natural phenomena. For example, changes in rain patterns, strong winds, the malnutrition of cattle and drying vegetation and water wells were the most commonly mentioned early warning signs (Table 3). Some early warning signs were prevalent in particular areas but missing in others. For instance, the withering of trees, drying up of rivers/streams and pollution of open water sources from strong winds gusts were observed only in the Highveld agro-ecological zone of Swaziland. Similarly, the growth of the traditional aloe plant, considered capable of drought prediction, was observed only in the Lowveld of Lesotho.

**Table 3:** Communities’ awareness of disaster early warnings.

	**Swaziland**							
	FGD1	FGD2	FGD3	FGD4	FGD5	FGD6	FGD7	FGD8
**Known disaster early warnings**								
Change in rain patterns: hail storms	√	√	√	√	√	X	√	X
Weakening of cattle	√	√	√	√	√	X	X	√
Consistent strong winds	√	√	√	√	√	X	√	X
Drying of grass and water wells	√	√	√	√	√	√	√	√
Pollution of open water sources from strong wind gusts	√	X	√	X	X	X	X	X
Through media (radio, TV)	X	√	√	X	√	√	√	√
Wind direction and/or shape of the Moon	X	X	√	√	√	√	X	X
Drying of trees, rivers and streams	X	√	X	X	X	X	X	X
Noisy exotic birds/frogs/grasshoppers/butterflies	X	X	√	√	X	X	√	X
A bumper fruit harvest in one season–drought	X	√	X	√	√	X	X	X
	**Lesotho**							
	FGD1	FGD1	FGD3	FGD4	FGD5	FGD6	FGD7	FGD8
Change in rain patterns: hail storms	√	√	√	√	√	√	√	√
Weakening of cattle	√	√	√	√	√	√	√	√
Consistent strong winds	X	√	√	√	√	√	√	√
Drying of grass and water wells	X	√	X	√	√	√	X	X
Pollution of open water sources from strong winds gusts	X	√	X	X	X	√	X	X
Through media (radio, TV)	X	√	√	X	√	√	√	X
Government warning via SMS	X	√	√	X	X	X	X	X
Change of wind direction from south to north	X	√	√	√	√	√	√	√
Presence of butterflies destroying crops	X	√	X	X	√	X	√	√
Growth of traditional aloe plant	X	√	X	X	X	X	√	X

√ consistently mentioned by many people; X never mentioned. **Swaziland study areas**: Mpolonjeni, Maphalaleni, Nkilongo. **Lesotho study areas**: Mpharene, Ramarumo, Kubake, Malumeng.

Other early warnings based on observation of the environment and natural phenomena included the excessive breeding of butterflies, changes in the Moon’s shape, noisy and exotic birds, noisy frogs and changes in wind direction, which were linked to disaster prediction, as represented in the statements below.

*[W]hen we see a heavy presence of butterflies spoiling our fields and destroying our crops such as maize*, *sorghum*, *beans and pumpkins*, *or laying eggs*, *we know the drought is coming*.

FGD Lesotho

*If the rain does not come in August*, *it is another sign that there is going to be a bad harvest as the ground is very hard and difficult to plant*.

FGD Swaziland

*We dig portholes for conservation agriculture and when we see the ground is hard and dry*, *and the rain hasn’t come at the right time*, *we know that will not be a good year*. *In addition*, *there will be strong winds that blow away the clouds*, *we know there will not be any rain*, *it will be dry for a long time*.

FGD Lesotho

There was consensus regarding the importance of early warnings based on observation of the environment and natural phenomena across the study communities.

Communication of meaning: Participants suggested that early warning information based on their observation of natural phenomena was easily shared among themselves. Some participants said that they supplemented the information shared within their communities with scientific early warning information disseminated by government agencies. However, some FGD participants in both countries said that they either did not know about or did not receive early warning information disseminated by government agencies, which were too far away for many community members to seek information from them, as illustrated in the following:

*There is a challenge of government facilities being very far away*, *which hinders the process and information they come up with*.

FGD Lesotho

*We lack information and knowledge to be ready and prepared against the drought*.

FGD Swaziland

*We do not know what the government is supposed to do for us*, *we do not know where to find the information*.

FGD Lesotho

#### The application of appropriate actions

The term ‘appropriate actions’ is used here to mean the initiation of actions in response to drought effects. Giddens’ structuration theory posits that human action is driven by the power of execution and, in terms of resilience, both agency and structures are integral to community dynamics and the ability to withstand shocks. Our findings are categorised as follows.

Resolve to overcome adversity: Our study assessed communities’ ability to undertake activities that promote drought adaptation and resilience building. Some of the activities were common across FGDs in both countries, for example, the establishment of homestead gardens and small businesses, and external assistance (Table 4).

**Table 4:** Communities’ applied actions for drought adaptation and resilience building.

	**Swaziland**							
	FGD1	FGD2	FGD3	FGD4	FGD5	FGD6	FGD7	FGD8
**Short-term**								
External food assistance	√	X	√	√	X	√	√	√
Migration to work in mines in South Africa	X	X	X	X	√	X	X	X
Traditional practices/rituals	√	X	√	X	X	X	√	√
Casual jobs	√	X	X	X	√	X	X	√
Selling livestock before they die	X	X	√	X	X	X	√	X
Reducing food rations	X	X	√	√	X	X	X	√
**Long-term**								
Homestead gardens	√	√	√	√	√	X	√	X
Savings groups	X	√	X	√	√	√	X	√
Rearing of goats, sheep, pigs and poultry	X	X	X	X	√	X	X	√
Crop diversification	X	√	X	√	√	X	√	X
Food stocks (reserves)	X	√	X	√	√	√	X	X
Conservation agriculture	√	X	X	X	X	X	X	X
Water harvest/building water dams/reservoirs	X	√	X	X	X	√	X	X
Looking for off-farm employment	X	X	X	X	√	X	X	X
Reusing wastewater for gardens	√	√	X	X	X	√	X	X
Establishing small businesses	√	√	X	X	√	√	X	√
	**Lesotho**							
	FGD1	FGD1	FGD3	FGD4	FGD5	FGD6	FGD7	FGD8
**Short-term**								
External food assistance	√	√	√	√	√	√	√	√
Community members helping each other	X	X	X	X	√	√	√	X
Migration to work in mines in South Africa	X	X	X	√	X	√	X	X
Traditional practices/rituals	√	√	√	√	√	√	√	√
Casual jobs	X	X	X	X	X	√	X	X
Community prayer meetings	X	X	√	√	X	√	X	X
**Long-term**								
Homestead gardens	√	√	√	√	√	√	√	√
Savings groups	√	√	√	√	X	X	√	√
Rearing goats, sheep, pigs and poultry	√	√	√	√	√	√	√	√
Crop diversification	√	√	X	X	√	X	X	√
Food stocks (reserves)	X	X	X	X	√	√	√	√
Conservation agriculture	√	√	√	√	√	√	√	√
Water harvest/building water dams/reservoirs	√	√	√	√	√	√	√	√
Looking for off-farm employment	√	X	X	√	X	X	X	X
Reusing wastewater for gardens	√	√	√	√	√	√	√	√
Establishing small businesses	√	√	√	√	√	√	√	√

√ consistently mentioned by many people; X never mentioned. **Swaziland study areas**: Mpolonjeni, Maphalaleni, Nkilongo. **Lesotho study areas**: Mpharene, Ramarumo, Kubake, Malumeng.

The findings show changes in farmers’ behaviour. For instance, FGD participants in four out of the eight FGDs in each country indicated that they had diversified their agricultural crops from the preferred and widespread growing of maize to short-maturing crops and vegetables, and they also practised conservation farming as an important strategy (Table 4). However, access to markets for their produce was a barrier, especially for farmers who sought to produce for home consumption as well as the market:

*We establish homestead gardens as a response to a disaster*. *We grow fruits and vegetables*, *but we have no markets for our produce*. *We could do better if we find support with identifying markets for our produce and the transport of the produce to the markets*.

FGD Lesotho

*During the drought*, *we produce vegetables as a coping mechanism*, *but we cannot find a market to sell our produce*. *We would have liked [… to work with us] to identify markets for our vegetables*.

FGD Swaziland

Interestingly, all FGD participants in Lesotho stated that they had established home gardens, while only participants in six out of the eight FGDs in Swaziland acknowledged having established home gardens (Table 4).

Our findings show differences between cattle farmers in Lesotho and Swaziland. In Lesotho, there was no evidence of farmers destocking. In Swaziland, some FGD participants suggested that livestock farmers destocked before a drought and this enabled them to receive good value for their cattle (Table 4). Those who did not destock had to sell their cattle when prices were low and some cattle had already died:

*[NGOs] trained us on how to sell livestock before they die and keep the money in the bank for use in difficult times*, *how to reduce the amount of foods consumed and food wastage*.

FGD Swaziland

Our findings show the affected communities were resourceful in identifying alternative sources of income. This was evident in the ability of able-bodied men to seek employment opportunities, especially in the mines in neighbouring South Africa, and remit income to their households:

*When there is no rain*, *there is nothing we can do*. *We migrate to South Africa in search of employment in the mines*. *When the conditions at home improve*, *we return and home to farm*.

FGD Lesotho

*[W]e stop selling produce and stock surplus for use in bad times or we migrate to South Africa to work in mines*.

FGD Swaziland

Other activities identified included taking casual jobs, community members helping each other, and rearing drought-resilient livestock such as goats, sheep and poultry as alternatives to cattle, which do not cope well in drought conditions.

There was evidence of farmers acquiring new non-agricultural knowledge and skills to diversify their livelihoods. For example, FGD participants had acquired a variety of skills, such as skills in the construction of basic infrastructure and processing of local products, to supplement their household incomes:

*[They] trained us in Vaseline production and built sanitary pits*, *and provided us with sewing machines … These skills will help us cope with and bounce back from disasters*.

FGD Lesotho

*We have groups that produce floor polish*, *we are ready and trained*. *They have taught us not to rely on external assistance through training to help us become entrepreneurial and business-oriented*. *We will be able to sustain ourselves … This will help us bounce back from disasters*.

FGD Swaziland

Community and institutional support: Some FGD participants credited their adaptation and resilience activities to NGO (non-government organisation) interventions such as training and capacity building, and mobilisation into groups. The participants acknowledged that their participation in training about food storage, pasture regeneration and saving, and their membership of farmer producer groups, empowered them to be active participants in resilience building, to grow their small businesses and to recover to better conditions than their pre-drought state:

*[We] established groups of vegetable producers and farmer associations [and] we trained in pasture regeneration for livestock*. *These skills will help us bounce back*.

FGD Lesotho

*We have learned how to stock food for use in difficult times and better management of food portions and to reduce food wastages*.

FGD Swaziland

However, the study results show that the effects of these interventions were on a small scale and did not cover all drought-affected communities:

*They [NGOs] taught us how not to rely on external assistance through training to help us become entrepreneurial and business-oriented*. *They have taught us how to use drought-resistant crops such as sorghum*, *cotton*, *beans*. *However*, *the challenge is not all community members belong to these initiatives*, *hence generating jealousy and conflicts*. *Those in savings group can buy whatever they want*, *hence some people think … [they are] favouring certain members of the community*, *which are wrong assumptions*.

FGD Swaziland

*Those [NGOs] will leave a long-lasting legacy behind*, *such as assets*, *saving groups and skills such as farming skills*. *But what has been neglected is the youth*, *who are excluded from initiatives and not engaged in programmes*.

FGD Swaziland

*Even if they [NGOs] leave*, *communities have been capacitated*, *the community is food-secure and resilient from any environmental shock or stress*.

FGD Lesotho

Our findings suggest that the governments of both countries and NGOs intervened with assistance such as food and water. However, some FGD participants suggested that this externally generated assistance deprived them of their self-esteem and encouraged laziness among some community members:

*There is too much reliance on external assistance in our community*. *Community members are [too] lazy to even implement their own programmes if they are supported to improve their livelihoods*, *and this is complicated by poor follow-up by the government and NGOs*.

FGD Lesotho

*It is painful to rely on external assistance*. *We have everything*, *but we are powerless on harnessing the community strengths and resources*.

FGD Swaziland

Understanding of abstract concepts: Some participants linked disasters to the supernatural and suggested that they require supernatural solutions. Such solutions can be divided into two main forms of religious activity, namely, ritualistic supplication performed by traditionalists and prayer events. Community leaders organised these events separately based on belief. Some FGD participants in Lesotho said they used prayer to invoke divine intervention to end drought. Other FGD participants in both countries suggested they participated in traditional rituals to appease the spiritual realm and end the drought or mitigate disaster impacts:

*We do some rituals to respond to or mitigate a disaster*. *We mix ash*, *salt and water*, *and throw the blend in the direction of the wind to stop a storm*.

FGD Swaziland

*Community members attend prayers meetings organised by the chief and hope for the best*.

FGD Lesotho

Rituals were also performed to induce rain and end drought spells, as expressed in the following response:

*We take a special tree to the river and do some rituals to bring back the rain*.

FGD Swaziland

FGD participants suggested that adversity, especially disasters, fostered participation and social support among communities. The recurrent nature of disasters, especially droughts, had led to support systems of collective innovation, such as self-help initiatives that supported members in times of need:

*We have been trained … on small businesses*, *income generating and financial management … [which has] allowed us to form savings groups*, *but members are self-selected based on interest*.

FGD Swaziland

*We formed associations to support each other with seeds and manure for food production*, *[and] savings groups where individuals can borrow and save with interest*.

FGD Lesotho

Exercise of power and control: Interestingly, the exercise of power manifested through household decision-making and resource allocation and control. Female participants suggested that men used household income on non-necessities. When men were in charge of money, they did not involve their spouses in deciding how the money should be used. When the women earned their own money, they learned not to declare it to their male spouses and to spend it quickly on household needs, such as food, before their husbands had the chance to take it:

*We have formed some savings groups and this helps us generate money to pay school fees … medical care*, *establish small businesses and to meet household needs such as buying corrugated iron*, *nice clothes and eat pizza … we earn money but our husbands do not*. *So we have somewhat control over our money*, *but we do tell them after expenditure so they do not take our money away before it is put to use*. *This leads to marital conflicts*.

FGD Swaziland

*[Most] initiatives target women and men are left out*. *Only women get empowered and men should be a priority too*. *However*, *women get targeted because they are more proactive*, *while men are lazy and do not attend meetings*. *Those flourishing are widows or single mothers*.

FGD Swaziland

Additionally, the exercise of power was noted in donor and host governments’ control over NGOs’ operations and aid beneficiaries. FGD participants suggested that donors and host governments determined who should benefit among the affected communities and the type of assistance:

*It is the requirements for donors or government*, *which makes it difficult for selection criteria of people who need help*.

FGD Lesotho

*We get discriminated by government structures based on political orientation*. *Only those supporting certain political parties get help*. *The priority is to help us reorganise our own structures to better engage with the government structures*.

FGD Lesotho

*Donors send young people from America*. *They should settle within the community to see exactly the context in which the communities are living in and to understand their needs*, *but currently they are based in town*. *They come and do what they think is right and not exactly what the community is in need of*.

FGD Swaziland

People selected for the interventions remained on the list of beneficiaries, instead of rotating so that others equally affected could also participate as preferred by the FGD participants.

Determining work relations: Some FGD participants observed that people who organised themselves in groups, such as women’s groups, had a better chance of influencing and benefiting from institutional interventions than those who were not organised:

*[F]or people who have formed women’s group*, *there is a clear strategy and it becomes easier for the funding agencies to give those funds or donations*. *The funding is about promoting self-reliance*, *not dependence*.

FGD Lesotho

*The community need to have clearer structure so that when the donor comes*, *he will be able to know who to assist*, *because the donations are not going to be there forever*.

FGD Swaziland

### Resilience framework

**Fig 2:**
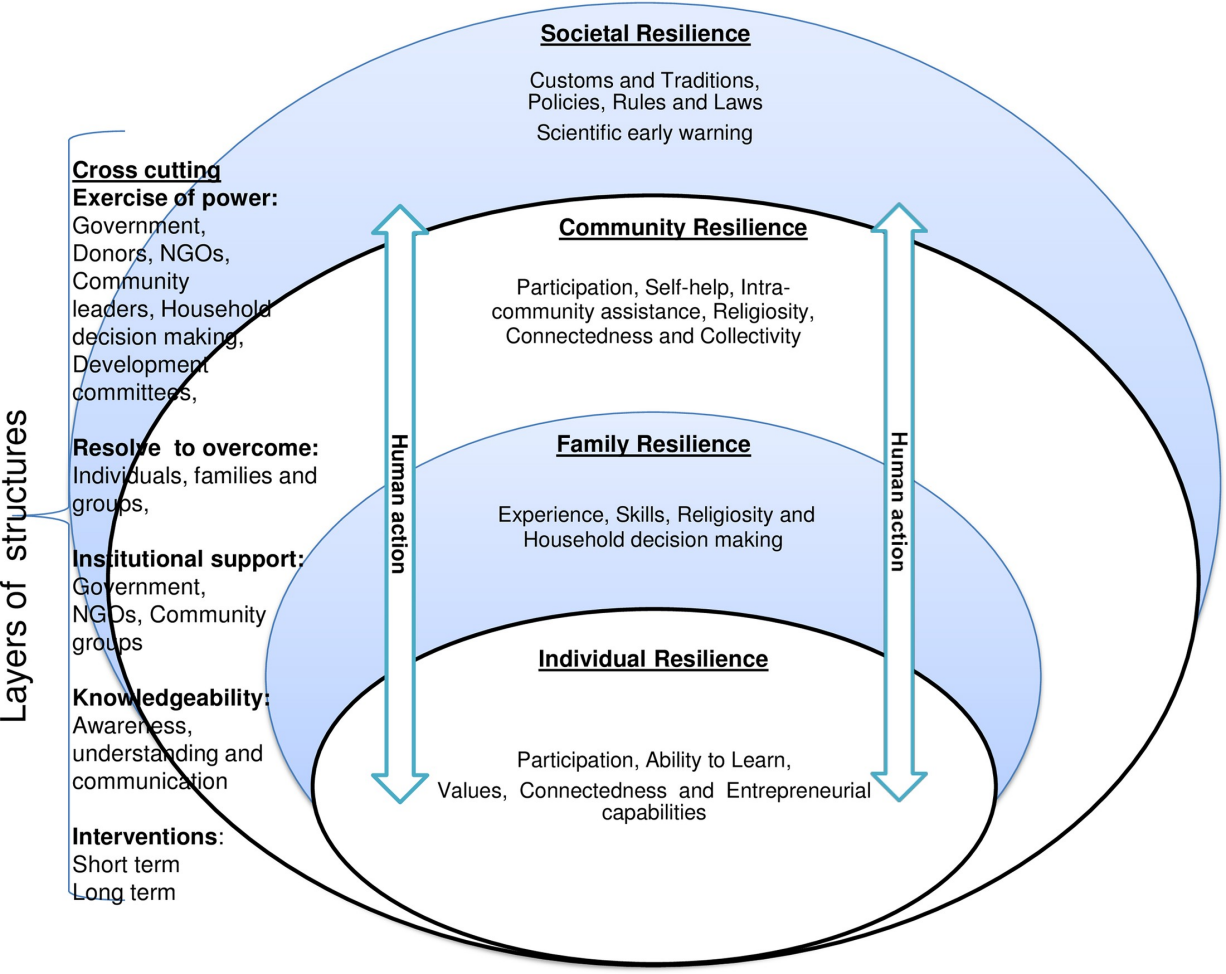
Resilience framework emerging from the study and informed by structuration theory.

Our findings, aided by structuration theory, have enabled the conceptualisation of a contextual resilience framework (Fig 2). The framework emerged after data analysis and during the processes of interpretation and description, which led to a line of thinking drawn from the interplay of communities’ actions and the harsh environment, and the subsequent actions and reactions. The emergence of human action as a primary construct of the framework is consistent with the study findings, which pivot around communities’ actions in line with structuration theory. Using the study findings and drawing from structuration theory to inform the framework are consistent with the study method and have been previously documented [75].

In this framework, the exercise of power, institutional support, communities’ inherent knowledgeability and their resolve to overcome drought effects cut across the different levels of society. This collectivism implies that, for sustainable, long-term resilience, all facets of society should be engaged. The framework recognises the complementary roles of short-term and long-term interventions in building resilient communities. We posit that this framework reflects the collectivist culture of the study population and acknowledges individuals as human agents acting alone and/or nested within families, communities and the greater societal structures. The framework is contextually and culturally appropriate, and presents a multifaceted view of resilience while providing a broad spectrum of options. It also offers a basis upon which to develop context-specific indicators to benchmark resilience.

## Discussion

We have used structuration theory to critically examine the influence of recurrent drought on the resilience of drought-prone communities. The theory has enabled our understanding of communities’ actions, reactions, knowledge and experience through the analysis of agency and structure in the study communities. FGD participants perceived resilience through the understanding of early warnings and the application of appropriate actions to cope with, adapt to and overcome drought.

### Early warnings

Consistent with structuration theory, the findings suggest that the most common early warning signs were based on communities’ knowledgeability and experience in observing and interpreting changes in the environment. However, the early warning signs varied between FGD participants. This could be a reflection of the differences between the agro-ecological zones. Therefore, the existence of an early warning sign in one agro-ecological zone may not have the same meaning in another zone. Such inconsistencies could mislead farmers into preparing for a disaster that does not occur or ignoring the early warnings and experiencing the full brunt of a disaster. Notably, there was no intuitional support to harness communities’ knowledgeability and experience, which informed the most common form of early warning. This reflects the power of institutions such as NGOs to selectively choose interventions that align with their objectives, as opposed to strengthening communities’ inherent capabilities. However, evidence suggests that communities’ knowledgeability and experience in relation to predicting disasters have been widely acknowledged in other parts of the world [76–78].

There were different perspectives among FGD participants on whether they received and used scientific early warnings. Some participants affirmed that they received the government-disseminated early warnings; others highlighted that they received early warning information too late in the agricultural season to use the information. Still other FGD participants said they did not receive the information at all. More farmers in Lesotho did not receive early warnings than their counterparts in Swaziland. The farmers who received early warning information did so through the media and mobile phone short messaging services (SMS). Evidence suggests that NDMAs receive early warnings from the SADC regional early warning mechanisms and national vulnerability assessment committees, and coordinate commensurate activities [27, 29]. The inconsistencies reflect gaps in early warning dissemination strategies and show that power and control over resources lie within institutions and those they are accountable to, as opposed to the communities they serve. However, previous studies in other regions established that poor dissemination of scientific early warnings weakens communities’ ability to mitigate hazard impacts and build resilience [38].

### Application of appropriate actions

Consistent with structuration theory, institutions such as government agencies and NGOs play an important role in supporting communities to cope and adapt. For example, the study results suggest that communities relied on external assistance in the form of emergency relief during drought-induced disasters. The assistance came in different forms, such as food and water aid, as well as livestock fodder assistance. While such interventions stave off hunger in times of crisis, they do not address salient vulnerabilities, such as environmental degradation, poverty and poor macro planning, that aggravate drought [40, 79]. These interventions enable affected communities to withstand and go through a drought but without improving their capabilities. As such, the communities remain in the same state as prior to the disaster. Therefore, it is not surprising that study participants observed that free assistance had inadvertent negative outcomes and was a barrier to self-help initiatives and self-actualisation. Nonetheless, it would be myopic to perceive this as a community weakness in the context of recurrent drought. Communities share the responsibility for development and improvement with their governments. Countries like Australia experience intense and prolonged droughts with devastating consequences for farming. However, their strong economy, governance structures, ingenuity, good health systems and social capital sustain farming communities [80–82].

Drought-induced food insecurity in Lesotho and Swaziland has existed for over three decades, which implies that food shortages are anticipated [40, 83]. The situation is aggravated by the AIDS burden that has plagued the two countries since the 1980s. Some scholars have labelled the complex mixture of drought, HIV/AIDS pandemic and food insecurity as a new type of famine, even though the situation does not meet the famine thresholds [84]. Needless to say, timely external assistance has been pivotal in cushioning both countries from famine, acute malnutrition and hunger-induced mortality as experienced in other sub-Saharan countries [85, 86]. Both Lesotho and Swaziland have continuously and successfully outsourced their disaster response to the international community [40]. While the outsourcing is interventionist in nature, it has prevented desperation and made timely humanitarian aid an important aspect of drought resilience. Whether aid will remain timely and sustainable over time as drought-induced disasters become more frequent remains a subject for further investigation. Nonetheless, it is important that communities’ views on this subject be considered in relation to the delivery of assistance.

Conversely, the inability of these countries to wean themselves from external humanitarian aid is a missed opportunity to build locally inspired disaster resilience. Building resilience to drought remains a critical concern and draws attention during and immediately after disaster occurrences [37, 86]. Thereafter it dissipates and remains only an internationally driven agenda. In Swaziland, resilience is promoted for adherence to international conventions such as the UN framework convention on climate change [87]. Similarly, dependence on international aid tilts the power balance in decision-making in favour of donor interests over those of the affected communities and the recipient governments. This approach absolves communities of critical decision-making responsibility and turns them into passive subjects, dominated by the choices and preferences of the international community. It therefore surrenders the building of resilience to drought to external forces, while inadvertently discouraging local ingenuity at the community and government levels. Holloway referred to this phenomenon as the externalisation of disaster management [40]. The international character of this phenomenon drives it to focus on international obligations, which may not necessarily be aligned with local needs and expectations.

Drought-induced disaster consequences in both Lesotho and Swaziland are severe. Yet we found some evidence of resilient behaviour among the study communities. For example, traditional maize and cattle farmers adapted to other non-farming livelihoods, such as micro-entrepreneurship, to diversify their incomes. Their scale of operations was sufficient for some households, but not significant enough to effect change across communities. Similarly, NGO interventions that sought to diversify livelihoods covered small populations in specific geographical areas. The gains were easily eroded when those they assisted failed to share their knowledge with the wider community. For example, the communities trained in the regeneration of livestock pasture did not stop the livestock of non-participating communities from grazing in the areas they had regenerated. The cultural understanding that land is communal means that communities traditionally share resources, which further degrades the gains made [88]. Unless such interventions are large-scale, the emerging pockets of resilience will be eroded. The erosion of such support increases community vulnerability and worsens conditions. Therefore, protection against the erosion of support efforts is an important feature of building resilience [49].

This study has found that institutional activities that contributed to the building of resilience prioritised specific groups, which is consistent with the concepts of determining relations of domination and the exercise of power. For example, women’s groups were prioritised for skills-related and microbusiness interventions. The acquired skills and microbusinesses empowered women to earn and manage their own incomes. They spent their own money without the knowledge of their husbands, as they feared the men would take and spend the money if they knew about it. While the empowerment of women is an aspiration of institutions like NGOs and governments, it upsets the traditional power balance and breeds household mistrust and conflict. Similar observations were made in Uganda, where women supported in a similar manner were considered too independent to remain good wives and were expelled from their marital homes [89]. Upsetting the household power balance subverts family cohesiveness, which is an important component of resilience.

In line with structuration theory, the spirituality of the study communities was influential in community understanding of abstract concepts. They associated disasters with the spiritual world and were guided by this in their response to disasters. For example, people prayed and performed different rituals to appease the spiritual world and end droughts. While such beliefs and practices are generally accepted as providing psychological satisfaction, their contribution to ending drought is minimal. However, our study has found that they promote unity and cohesion, which are important elements of resilience. This is consistent with earlier studies in different regions which found that shared belief systems were pivotal in collective responses and the building of long-term resilience to disasters [76, 90].

Migration to South Africa to search for work in the mines externalised redundant labour during drought. The incomes from this externalised community labour were used to cover family expenses such as food, health care and education for children. This finding is consistent with other studies that show labour migration in times of adversity [91, 92]. However, structuration theory suggests that human actions and structures are integral aspects of communities’ ability to withstand drought and their continuity. Therefore, leaving affected communities in times of adversity further weakens their resilience. Moreover, the trend of migration to work in South African mines has poor health and well-being outcomes, such as the spread of infectious diseases and the breakdown of family cohesion associated with prolonged spousal or parental absence from the family [92]. In addition, increased occurrence of drought will lead to large-scale migration if resilience interventions are not urgently scaled up [91].

### Limitations

The study was based on responses by FGD participants drawn from local social networks which may have limited diversity. Senior government units of administration were not represented. In addition, we did not segregate FGD participants based on their skills or level of education, which may have influenced varying levels of perception and reflection. There was a higher representation of female than male participants in the study; however, this is consistent with the existing community social groups that participants were drawn from. Conversely, the findings expressed in this study are only representative of communities’ perceptions, and not necessarily scientific consensus.

### Policy implications

Our findings suggest the need for policy interventions that emphasise inherent resilience capabilities in disaster management initiatives. This can be achieved through the promotion of knowledgeability, value systems, resources, connectedness, participation, decision-making and collectivism, all of which constitute communities’ social capital. It is through this social capital that communities conceptualise their understanding, initiate actions and embrace support systems. Interventions seeking to build and strengthen resilience should utilise these transformative capabilities to encourage the active participation of communities and ensure that inherent capabilities are not disregarded.

Both Lesotho and Swaziland are beneficiaries of the well-established SADC early warning system that has been instrumental in enhancing member states’ disaster management capabilities. Nonetheless, there is need for further research on the gaps in the dissemination of early warnings among rural communities. There is a need to support and link locally understood early warnings to national systems in order to create integrated early warning systems. This can be achieved through formal recognition and support of traditional knowledge, which will ultimately increase communities’ participation, and improve the communication and dissemination of information between government and communities.

Our results also show evidence of subsistence farmers acquiring new non-agricultural knowledge and skills to diversify their livelihoods. However, these efforts were limited by the scale of interventions, the government and donors’ control. Therefore, the interventions benefited small proportions of the communities, even though most of the rural populations were farmers affected by recurring drought. For resilience interventions to reach more people, they must be integrated within existing government sectors such as health, education, infrastructure and agriculture. For this to be achieved, resilience has to be embedded within national development planning. Notwithstanding this planning, interventions should actively promote participation such as the delegation of authority to host communities, especially in critical decision-making including resource allocation. Care must be undertaken to ensure participation is genuine, and not tokenistic. Furthermore, disaster relief assistance should be considered in contexts where it is the only relevant intervention and should be tied to specific resilience benchmarks.

## Conclusion

Consistent with structuration theory, this study validates resilience as a construct of the interaction between human action and social structures, especially through institutional support, resolve to overcome adversity, knowledgeability and work relations. We have established that community actions were inspired by their experience of drought and furthered by institutional support. However, the exercise of power and control, especially by donors, was a barrier to drought resilience activities. The effectiveness of institutional support hinged on harnessing communities’ social capital.

## Supporting information

S1 Appendix FGD guide—English version. (DOCX)

S2 Appendix FGD guide—Siswati. (DOCX)

S3 Appendix FGD guide—Sesotho. (DOCX)
