# Supplementary material for: Understanding disaster resilience in communities affected by recurrent drought in Lesotho and Swaziland—A qualitative study
Source: PLoS One. 2019 Mar 1;14(3):e0212994. doi: 10.1371/journal.pone.0212994 (PMC6396921; doi:10.1371/journal.pone.0212994)
Supplement: S1 Appendix — (DOCX) [file pone.0212994.s001.docx]

**S1. Appendix: Focus Group Discussion Guide- English**

1. **Introduction:**

Welcome participants and introduce yourself, and the note taker.

Consider the following:

- Who we are and why we are conducting the FGD
- Why we need your participation and how the information gathered will be handled
- The purpose of the discussion is to assess how World Vision has supported the communities to overcome recurrent drought impacts and the way forward.
- You may withdraw your participation or refuse to answer questions you feel uncomfortable with at any time.
- Any information discussed here today will be confidential, and your name will not be associated with anything you say during our discussion.
- Do not to repeat the discussion after we have left and to respect each other’s confidentiality.
- We request your permission to record the discussions so that we capture the richness of the discussions.
- Please feel free to walk away now if you disagree with the above, if you agree, please write down your name, gender and occupation on the list.

1. **Ground Rules**

Request to brainstorm the ground rules and write down their responses. Ensure the following are included:

- All ideas are important and valuable
- Respect each other’s opinion
- One person speaks at a time and do not talk over each other or cut off the person before they complete their point.
- Information provided in the focus group must be kept confidential
- Everyone should contribute
- There is no wrong or right responses, all ideas are valuable.

Ask if the group has any questions before you start and address them accordingly.

1. **Begin the FGD with Introductions**

- Everyone to introduce themselves.
- Turn on Tape Recorder

*Note: Give people time to think before answering the questions and don’t move too quickly. Probe to elicit exhaustive responses, but move on when you begin to hear repetitive information.*

**Questions:**

1. In this area, how do people know that a natural disaster is going to happen?
2. When people in this area realise they are at risk of a natural disaster, what do they do? What kind of disaster plans do they have? How did these plans come about?
3. How has your community actively prepared for disasters? (Probe: what community structures or resources have facilitated this? What worked? What has not worked? Are you involved in decision making?
4. What types of disasters has your community experienced over the last three years? (probe: how many families were affected by these disasters and how?)
5. What measures have the people in your community undertaken to bounce back from a disaster? (Probe: what would an ideal recovery entail? What lessons from the past disasters have your community implemented?)
6. When responding to disasters, what do you see as the main barrier(s) to collaboration between your community and i) local government? ii) NGOs
7. Could you please provide an example of an obstacle or negative experience you have had when collaborating or trying to collaborate with the i) local government, ii) NGOs in your areas.

This is the end of our discussion, thank you for sharing your ideas and opinions. If you have additional information that you did not get to say in the focus group, please feel free to come and talk to any one of us before we leave.

1. **Materials and supplies for focus groups**

- FGD guide
- A tape recorder
- Notebook for note-taking
- Participants list
