# Supplementary material for: Understanding disaster resilience in communities affected by recurrent drought in Lesotho and Swaziland—A qualitative study
Source: PLoS One. 2019 Mar 1;14(3):e0212994. doi: 10.1371/journal.pone.0212994 (PMC6396921; doi:10.1371/journal.pone.0212994)
Supplement: S2 Appendix — (DOCX) [file pone.0212994.s002.docx]

**Imigomo yetinkhulumo letolandzelwa- Siswati**

1. **Singeniso:**

Mukela labakhona utichaze wena nalotobe abhala.

Naka naku lokulandzelako:

- Kutsi sibobani nekutsi siyentelani lenkhulumo
- Sibudzingelani bukhona benu nekutsi lemininingwane letotfolakala itophatseka kanjani
- Inhloso yalentingcogco kubonisana kutsi World Vision uyelekelele njani imimmango kuncoba imitselela yesomiso kanye nenchubekela phambili.
- Ungaphuma kulenkhulumo nobe ungaphendvuli imibuto lengakuphatsi kahle.
- Konkhe lokutokhulunywa ngako lapha kutoba yimfinhlo, nekutsi libito lakho ngeke limbhandzakanywe nalebekukhulunywa ngako.
- Letindzaba atiphumeli ngaphandle tisala khona la, nihloniphe futsi netimfihlo talabanye.
- Sicela imvume yenu kutfwebula letinkhulumo sitogcina bungibo bato.
- Nikhululekile kusukuma nihambe nyalo nangabe ningavumelani nalolokulangetulu, nangabe niyavumelana nako sicela nibhale emabito, bulili nemsebenti leniwentako kuloluhla.

1. **Imigomo**

Cela kutsi nakhe imigomo lenitayilandzela niyibhale phansi. Cinisekisa kutsi lolokulandzelako kukhona:

- Yonkhe imibono ibalulekile futsi imcoka
- Hlonipha imibono yalabanye
- Kukhulume umuntfu munye ngasikhatsi sinye futsi ungamjubi lomunye nakasengakacedzi kukhuluma.
- Imininingwane yaletinkhulumo ayigcinwe iyimfihlo
- Wonkhe umuntfu akafake umbono
- Kute timphendvulo lekungasito naletingito, tonkhe tibalulekile.

Buta kutsi kute yini imibuto lekhona ningakacali, iphendvulwe ngendlela lefanele.

1. **Cala lenkhulumo ngekutsi natane**

- Nguloyo atisho kutsi ungubani.
- Vula umshini wekutfwebula

*Naka: Banike sikhatsi sekucabanga bangakaphendvuli, ungasheshisi. Butisisa kuciniseka kutsi utitfola tonkhe timphendvulo, kepha uchubeke nangabe sekuphindzeka lokukhulunyiwe*

**Imibuto:**

1. Kulommango wakini, bantfu bati ngani kutsi kutoba nesehlakalo semvelo?
2. Bantfu balommango nasebabona kutsi basengotini yesehlakalo semvelo, benta njani? Banatinhlelo tini letibukene netehlakalo? Letinhlelo tavela kanjani?
3. Ummango wakini wentani kulungiselela nekuhlangabetana naletehlakalo? (Butisisa: yini lokwentiwa ngummango kulungiselela kuhlangabetana naletehlakalo? Yini lokusebentile? Yini lokungakasebenti? Niyahlanganyela yini nakutsatfwa tincumo?
4. Nhloboni yetehlakalo ummango wakini loke wahlangabetana nato kuleminyaka lemitsatfu leyengcile? (Butisisa: mingakhi imindeni leyatsintseka kuletehlakalo, yatsintseka kanjani?)
5. Ngutiphi tindlela bantfu lebatisebentisile kubuyela esimeni? (Butisisa: kubuyela esimeni lesikahle kungadzingeka kwentiweni? Kusukela kuletehlakalo letengcile ngutiphi tifundvo lenitisebentisile)
6. Maniphunga tehlakalo yini lenikubona kutingcinamba ekusebentisaneni emkhatsini wemmango na 1) hulumende 2) tinhlangano letitimele
7. Yini tibonelo tetikinga lenike nahlangabetana nato nanisasebentisana nobe nisazama kusebentisana na 1) hulumende 2) tinhlangano letitimele emimangweni yenu

Siyabonga kusinika imicabango nemibono yenu. Nangabe kukhona lokusele sisakhulumisana khululeka kukhuluma namunye wetfu sisengakahambi. Sekuphela kwato letinkhulumo tetfu.

1. **Tintfo letitodzingeka**

- Imigomo yetinkhulumo letolandzelwa
- Umshini wekutfwebula
- Libhuku lekubhalela
- Luhla lwalabakhona
