# Supplementary material for: Understanding disaster resilience in communities affected by recurrent drought in Lesotho and Swaziland—A qualitative study
Source: PLoS One. 2019 Mar 1;14(3):e0212994. doi: 10.1371/journal.pone.0212994 (PMC6396921; doi:10.1371/journal.pone.0212994)
Supplement: S3 Appendix — (DOCX) [file pone.0212994.s003.docx]

**Tataiso ea ho tsamaisa lipuisano tsa lihlopha tse hloailoeng tsa batho- Sesotho**

1. **Selelekela**

Lumelisa ba nkang karolo boithutong bona, itsebise bona, uena mmoho le ea ngolang litaba tsa boithuto.

Ela hloko tse latelang

- Na re bo mang le mabaka ao re etsang boithuto bona sehlopheng sena se hloailoeng
- Mabaka ao re hlokang tlatsetso ea lona ka oona, le ka moo litaba tse bokeletsoeng mona li tla sebelisoa ka teng.
- Totobatsa hore sepheo sa lipuisano tsena ke ho ithuta hore na World Vision e t’sehelitse sechaba joang ho fenya komello le litlamorao tsa eona, le hore na ho tla tsoeloapele joang)
- U na le tokelo ea ho ikhula lipuisanong tsena kapa ho hana ho araba lipotso tseo u ka utloang li sa u t’soare hantle neng kapa neng.
- Litaba tse builoeng mona tsatsing lena li tla bolokoa ka lekunutu, le lebitso la hao le keke la amanngoa le tse t’sohliloeng nakong ea lipuisano tsa rona.
- Hoba re phethele lipuisano tsa rona ,elang hloko ho se li qoqe kapa ho li pheta kae kapa kae molemong oa ho bolokelana lekuntu le botsepehi.
- Re kopa tumello ea hore re hatise lipuisano tsa rona molemong oa ho re thusa ho fumana bohlokoa le botebo ba taba
- U bolokolohing ba ho ikhula le ho tsamaea haeba u sa lumellane le se koptjoang seratsoaneng se ka holimo. Haeba u lumela, re kopa u ngole tse latelang lethathamong le fanoeng mona: Lebitso la hao , na u Mme kapa Ntate (botona kapa botsehali) le hore na u sebetsang.

1. **Melooana ea tsebetso**

Kopa ho sekaseka melaoana ea tsebetso, ebe u ngola seo ho lumellanoeng ka sona. Etsa bonnete ba hore tse latelang li kenyelelitose:

- Maikutlo oohle a bohlokoa ‘me a bile a aheloa lesaka
- Ho hlompshoe maikutlo a bohle
- Ho buoe motho a le mong ka nako ‘me ho se kena-kenanoe le motho ea joalo kapa ho mokhaola ha a ntse a bua
- litaba tse fanoeng mona li bolokoe e le tsa lekunutu
- Boohle ba be le seabo
- Ha ho likarabo tse nepahetseng kapa ho fosahala ho feta tse ling, likarabo tsohle tsa fanoeng li bohlokoa ka ho t’soana .

Botsa hore na batho bana le lipotso pele u ka tsoela pele ho bua le bona

1. **Qala lipuisano ka ho itsebahatsa**

- Bohle ba ipolele/itsebise
- Bula sehatisa-mantsoe

*U fe batho monyetla oa ho inahana pele ba araba lipotso, ‘me u seke oa ba potlakisa. Etsa t’salo morao ea lipotso tsa hao moo u utloang u sa fumane likarabo tse hlakileng, empa u tsitlallele ho tsoele pele ka lipuisano haeba u belaela hore ho na pheta-pheto ea taba e tsoanang.*

**Lipotso**

1. Sebakeng sa lona moo, batho ba tseba joang ha koluoa ea thloleho e tlo etsahala?
2. Ha batho ba sebaka sa heno ba hlokomela hore ba tlokotsing ea koluoa ea thloleho ba etsa joang? Ba na le mefuta efe ea meralo ea likoluoa? Meralo e joalo e thehiloe/tlile joang?
3. Sechaba sa heno se sehlahlo ha kae boitukisetsong ba likoluoa? (tsoela pele ho botsa: na ke likomiti life sechabeng tse sebetsanang le taba ea boitukisetso ba likoluoa ‘me u be u fumane na be ithusitse joang ho fihlela sena ?, ke eng e shebahetseng e sebetsa? hape ke eng e sa sebetsang hantle? Na bana le seabo ho etsa liqeto/liqetong tse ba amang?)
4. Ke mefuta efe ea likoluoa e kileng ea oela sechaba sa heno nakong ea lilemo tse tharo tse fetileng? (Tsoela pele ho botsaKe malapa a makae a kileng a angoa ke likoluoa tse joalo, ‘me sesosa ene e le sefe?)
5. Sechaba sa heno se nkile mehato efe ho khutlisetsa maemo setloaeling? ( Tsoela pele ho botsa: Mehato e nepahetseng e kaba efe hore sechaba se boele se iphumane? Sechaba se ntlafalitse eng ka morao ho boithuto ho tsoa likoluoeng tse fetileng?)
6. Ha ho phalleloa likoluoeng, ke ntho life tseo le bonang li baka t’sitiso ea tsebelisano mmoho pakeng tsa sechaba le i) Puso ea Libaka? Ii) Mekhatlo e ikemetseng?
7. A’ku fane ka mohlala oa ntho e kileng ea baka t’sita kapa ho nyelisa tsebelisano mmoho pakeng tsa lona le i) Puso ea libaka ii) Mekhatlo e ikemetseng tikolohong ea lona?

Re leboha boitelo ba lona ba ho fana ka maikutlo . Haeba u kaba le litaba tseo u sitiloeng ho li bua nakong ea lipuisano le sehlopha, u bolokolohing ba ho atamela e mong oa rona pele re tsamaea. Re fihlile qetellong ea lipuisano tsa rona.

1. **Thepa le lisebelisoa sebakeng sa ho nt’setsa pele lipuisano tsa lihlopha tse hloailoeng tsa batho**

- Tataiso ea ho tsamaisa lipuisanolihlopheng tse hloailoeng
- Sehatisa-mantsoe
- Bukana ea ho ngola lipuisano
- Lenane la bankang-karolo c
